# FNIRS‐Based Energy Landscape Analysis to Signify Brain Activity Dynamics of Individuals With Depression

**DOI:** 10.1111/cns.70139

**Published:** 2024-12-01

**Authors:** Yushan Wu, Shi Qiao, Jitao Zhong, Lu Zhang, Juan Wang, Bin Hu, Hong Peng

**Affiliations:** ^1^ Gansu Provincial Key Laboratory of Wearable Computing, School of Information Science and Engineering Lanzhou University Lanzhou China; ^2^ Department of Psychological Medicine Seventh Medical Center of PLA General Hospital Beijing China; ^3^ Key Laboratory of Special Functional Materials and Structural Design, Ministry of Education Lanzhou University Lanzhou China

## Abstract

**Background:**

Major depressive disorder (MDD) is one of the most common mental disorders, and the number of individuals with MDD (MDDs) continues to increase. Therefore, there is an urgent need for an objective characterization and real‐time detection method for depression. Functional near‐infrared spectroscopy (fNIRS) is a non‐invasive tool, which is widely used in depression research. However, the process of how the brain activity of MDDs changes in response to external stimuli based on fNIRS signals is not yet clear.

**Method:**

Energy landscape (EL) can describe the brain dynamics under task conditions by assigning energy values to each state. The higher the energy value, the lower the probability of the state occurring. This study compares the EL features of 60 MDDs with 60 healthy controls (HCs).

**Results:**

Compared to HCs, MDDs have more local minima, smaller energy differences, smaller variations in basin sizes, and longer duration in the basin of global minimum (GM). The classification results indicate that using the four features above for depression detection yields an accuracy of 86.53%. Simultaneously, there are significant differences between the two groups in the duration of the major states.

**Conclusion:**

The dynamic brain networks of MDDs exhibit more constraints and lower degrees of freedom, which might be associated with depressive symptoms such as negative emotional bias and rumination. In addition, we also demonstrate the strong depression detection capability of EL features, providing a possibility for their application in clinical diagnosis.

## Graphical abstract

Our study uses energy landscape analysis to investigate the brain dynamics of patients with major depression (MDDs) under task conditions. The experiments show that brain networks in MDDs show more restraint and less freedom, which is associated with depressive symptoms such as negative emotional bias and rumination.

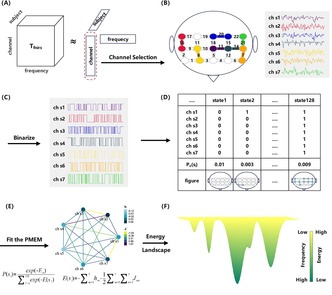

## 1 Introduction

Depression has become a significant public health concern, ranking as the second most prevalent human ailment, only behind heart disease [1]. According to statistics, there are approximately 350 million people globally suffering from depression [2]. However, the clinical detection rate of depression is less than one‐third [3]. The current diagnosis of major depressive disorder (MDD) is predominantly based on self‐assessment scales and clinical interviews, heavily reliant on the individual's subjective perception and the clinician's professional expertise [4]. Therefore, there is an urgent need to design more objective and effective assessment tools for MDD, thereby achieving auxiliary early screening for MDD.

Methods for disease screening based on physiological and behavioral signals have been extensively researched [5, 6, 7, 8, 9]. Physiological signals are difficult to disguise, making it easier to establish a mapping relationship between MDD's symptoms and signal indicators [10]. Functional near‐infrared spectroscopy (fNIRS) is an emerging brain imaging technique that possesses strong portability, real‐time monitoring, device portability, and cost‐effectiveness as advantages [11, 12, 13]. FNIRS can non‐invasively measure cortical hemodynamic changes, but it lacks specific biomarkers that accurately describe brain network activity [14].

Previous studies have demonstrated a link between the increased risk of MDD and difficulties in brain resource allocation when individuals face internal or external stimuli [15]. Specifically, this is characterized by decreased connectivity within the frontotemporal control region [16]. Additionally, impaired cooperation and synchronization between this control system and networks involved in internal and external attention are observed [15]. These alterations in network functions tend to sustain and exacerbate depressive symptoms, such as rumination, negative biases, and cognitive abnormalities [17]. Therefore, MDD can be regarded as a dysfunction in brain network functionality.

Currently, most brain network analyses based on fNIRS data utilize the functional connectivity (FC) analysis method. Chao et al. [18] found that compared to healthy controls (HCs), individuals with MDD (MDDs) exhibited abnormal FC of the bilateral ventrolateral prefrontal cortex (VLPFC) and bilateral dorsolateral prefrontal cortex (DLPFC). Dong et al. [19] assessed FC deviations in MDDs during cognitive tasks, observing reduced activation in the prefrontal cortex (PFC) of MDDs. FC analysis can reveal pairwise correlations between channels, providing valuable insights into the dynamics of brain networks and neural circuitry [19]. However, FC assumes that pairwise interactions between channels are independent of each other, potentially overlooking information related to higher‐order interactions [20]. For instance, the observed correlation between channel A and channel B in FC might be a combination of the correlations between channel A and channel C as well as between channel B and channel C [21]. For this issue, energy landscape (EL) analysis can serve as an alternative approach to FC analysis. The EL analysis embeds the pairwise maximum entropy model (pMEM), allowing estimation of large‐scale patterns of brain activity. In comparison with FC, pMEM has been shown to uncover more physiological information as it infers global activity patterns (i.e., activities across all channels) rather than independent pairwise interactions between channels [22].

In recent years, EL is predominantly applied for analyzing functional magnetic resonance imaging (fMRI) data, focusing on the spatial exploration of brain functional networks within specific regions of interest (ROI) [22, 23, 24]. ROIs are manually selected in these studies, potentially introducing human bias that could affect experimental consistency. In addition, EL is consistently utilized to quantify the intricate neural activity of the brain during resting state [25, 26, 27]. However, rest is an unconstrained state [28], which may make it difficult to capture the full extent of differences [29, 30]. Many studies involve emotional tasks to facilitate the activation of brain activity and induce emotional responses in participants [31, 32, 33, 34]. Greene et al. [35] pointed out that task states have greater research potential for brain networks compared to resting states and could be used to identify depression. Therefore, the application of traditional EL in understanding brain dynamics during task states remains relatively limited. Meanwhile, prior research has primarily focused on investigating brain functional impairments and cognitive disorders such as epilepsy [36] and Alzheimer's disease [37], lacking sufficient exploration into the brain's EL among individuals with mental disorders such as MDD.

To address the aforementioned issues, we propose a channel‐level data‐driven EL for the analysis of brain neural activity during task states in MDDs. Technically, a channel selection algorithm based on CANDECOMP/PARAFAC Decomposition (CPD) [38, 39] is utilized to choose seven channels. Subsequently, each channel signal is binarized using the mean signal value as a threshold. pMEM is then fitted to match the empirical data distribution of the binarized network states, ultimately constructing an EL. This approach establishes the most informative channel set instead of manually selecting ROIs, mitigating biases, and reducing redundant data. At the same time, our study provides a new perspective on task‐related EL analysis in MDDs.

Based on the proposed data‐driven approach, this study construct ELs using fNIRS data collected from 120 participants (60 MDDs and 60 HCs) under audio stimulation. The experimental results indicate that pMEM achieves excellent fitting of brain networks in both MDD and HC groups. The two groups share a pair of major states (0000111 and 1111000), but differences are observed in the duration within the basins of these major states, which are related to negative emotional bias and rumination. MDDs exhibit an increase in the number of LMs and the duration of the GM, along with a decrease in the standard deviation of basin sizes and the energy difference. We further demonstrate that EL features can be used for depression detection. These findings suggest the presence of network abnormalities in MDD, typically characterized by more constraints and lower degrees of freedom.

## 2 Paradigm and Data

### 2.1 Participants

By means of rigorous screening, meticulous matching, and efficient data organization, 120 participants took part in this research, comprising 60 MDDs and 60 HCs. All participants recruited for this study were screened by psychiatrists from the Department of Psychiatry at the Third Hospital of Tianshui, Gansu, China, using the Mini International Neuropsychiatric Interview (M.I.N.I) [40] and the Mini‐Mental State Examination (MMSE) [41]. The 9‐item Patient Health Questionnaire (PHQ‐9) [42] and 17‐item Hamilton Depression Scale (HAM‐D‐17) [43] scale were utilized to measure the severity of depression in the MDDs. This experiment received approval from the local research ethics committee and obtained written informed consent from all participants after explaining the experimental paradigm.

All participants are between the ages of 18 and 60, have not taken any psychotropic drug or prescribed controlled substances in the past 2 weeks, have no brain damage, epilepsy, or severe physical illnesses, and are not pregnant or lactating. MDDs are diagnosed using the M.I.N.I and MMSE by professional doctors, with a PHQ‐9 score ≥ 10 and HAM‐D‐17 score ≥ 17. Only MDDs meeting one of the following two conditions are selected: (1) first episode of depression, with no history of self‐medication prior to this; (2) recurrent episodes, but the previous episode has concluded treatment, and no medication has been taken for at least 6 months prior to this consultation. MDDs have no comorbidities of schizophrenia, anxiety, or other mental disorders, and they do not exhibit high‐risk suicidal tendencies. HCs have no history of mental health issues in themselves or within the family, with a PHQ‐9 score ≤ 4 and HAM‐D‐17 score ≤ 7.

The independent‐sample *t*‐test is used to assess the differences in age, PHQ‐9, and HAM‐D‐17 between groups, while the chi‐square test is used to evaluate gender differences between groups. Table 1 shows the statistical results for clinical features, indicating no statistical differences between the two groups in terms of gender and age. The MDD group has higher PHQ‐9 and HAM‐D‐17 scores compared to the HC group. Hence, we disregard the potential effects of age and gender in the subsequent experiments.

**TABLE 1:** Clinical characteristics of MDDs and HCs.

Characteristics	MDDs	HCs	*p*
Age (years)	37.63 ± 13.99	36.95 ± 13.18	0.81
PHQ‐9	15.48 ± 4.94	1.43 ± 1.61	0.00
HAM‐D‐17	27.25 ± 5.66	4.27 ± 2.50	0.00
Gender (male/female)	30/30	30 / 30	1.00

*Note:* The independent‐sample *t*‐test and the chi‐square test are used to compare the clinical characteristics between the MDDs and HCs.

Abbreviations: HCs, healthy controls; MDDs, individuals with MDD.

### 2.2 Paradigm

The use of audio stimuli as a simple and effective method for inducing emotions has found wide application in the fields of fNIRS and affective computing [44, 45]. We design a paradigm based on audio stimuli, consisting of four blocks, each comprised of four types of trials: happy, calm, fear, and white noise. The 16 trials are arranged using a Latin square design to reduce the impact of sequence order on the experiment. Each trial lasts for 18 s, with a 20‐s rest period between two trials to facilitate the restoration of the hemodynamic response to its baseline level. The entire experiment takes approximately 15 min. The experiment takes place in a quiet room, and participants are instructed to keep their eyes closed and maintain bodily stillness throughout the entire experimental procedure. Figure S1 in the Supporting Information depicts the overall flow of the paradigm.

### 2.3 Data Acquisition and Preprocessing

In accordance with the 10–20 international system, the fNIRS system is configured with 22 channels in the prefrontal cortex region for this study. Figure S2 in the Supporting Information illustrates the electrode arrangement of the fNIRS system. Channels 2, 7, 9, 14, 16, 21, and 22 are located in the superior frontal gyrus (SFG) region, while the remaining channels are situated in the middle frontal gyrus (MFG) region [46]. The experiment gathers all fNIRS signals utilizing a multi‐channel continuous‐wave fNIRS system (NIRx Medical Technologies LLC) at a sampling rate of 7.81 Hz. The NIRStar data acquisition software (version 15.1) is used to document the configuration of the optode placement. The raw signal from fNIRS consists of the time series of optical intensity values at two wavelengths, 760 and 850 nm, for each channel. The modified Beer–Lambert law (MBLL) is utilized to compute the concentrations of oxygenated hemoglobin (HbO), deoxygenated hemoglobin (HbR), and total hemoglobin (HbT) corresponding to the optical intensity data [47].

Then, we categorize the fNIRS signals into positive (happy), neutral (calm), negative (fear), and all stimuli based on the types of stimulus tasks. This further segmentation aids in a more detailed exploration of how different emotional stimuli impact MDDs. Therefore, the fNIRS signals are divided into four sequences.

Hemodynamic signals unavoidably contain various sources of interference, such as experimental noise, physiological noise, and instrument noise [48, 49]. The Homer2 toolbox [50] in MATLAB 2019a can be used for preprocessing. The hmrMotionArtifactByChannel function recognizes motion artifacts in the time periods, with specific parameters set as follows: AMPthresh = 5.0, tMotion = 0.5, STDEVthresh = 20.0, and tMask = 3.0 [18]. The hmrMotionCorrectSpline function with pSpline = 0.99 performs correction. Additionally, the hmrBandpassFilt function with a cutoff frequency of 0.01 to 0.2 Hz is applied to remove heartbeats (> 1 Hz), respiration (0.2~0.5 Hz), and high‐frequency noise [51].

## 3 Method

**FIGURE 1:**
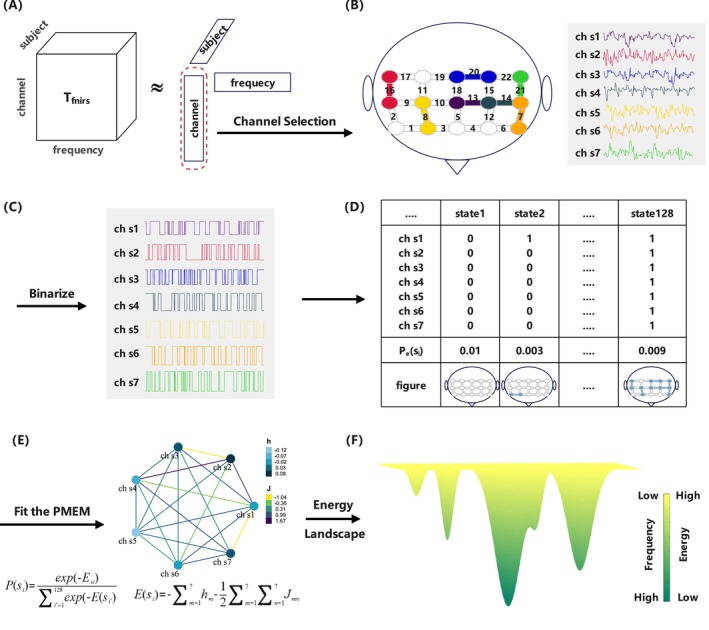
The flow chart of EL analysis. (A) fNIRS data is organized as a three‐dimensional tensor, with the three dimensions representing channel, frequency, and subject. Among these, the channel dimension requires further selection. (B) Seven channels are selected through the CPD algorithm and Pearson correlation analysis. (C) Signal binarization. Data points above the mean are marked as 1, while those below are marked as 0. (D) Each column represents the 7‐digit code corresponding to a state, and the empirical appearance probability is calculated. (E) A pairwise maximum entropy model is simulated to obtain energy corresponding to each state. (F) Energy landscape model schematic. The higher the energy of a state, the lower its frequency of appearance.

We analyze the EL of task‐state fNIRS signals to explore the physiological differences in response to external emotional stimuli between MDDs and HCs. The flowchart of the EL analysis in this study is illustrated in Figure 1. The framework mainly comprises four steps: channel selection, signal binarization, fitting the pMEM model, and constructing the EL. Firstly, the preprocessed data are structured into a three‐dimensional tensor, with dimensions representing channels, participants, and signal frequency, respectively. The channel factor matrix is extracted by CPD and utilized for channel selection. To better capture brain activity patterns [21], we binarize the continuous fNIRS signals. Following this, the relative frequency of occurrence for each state is computed. Subsequently, the pseudo‐likelihood maximization method is employed to estimate the pMEM model, with accuracy index r serving as the model evaluation metric. Finally, the EL is plotted using the energy of each state, and multiple EL features are extracted to compare the differences and similarities between MDDs and HCs.

The EL conceptualizes brain signals as a network composed of various states, each characterized by an energy value inversely related to its probability of occurrence [37]. Consequently, states with lower energy levels are more likely to occur. Due to varying energy levels among different states, there emerge unstable energy peaks (with low probabilities of occurrence) and stable energy basins (with high probabilities of occurrence), creating distinct energy constraints [52]. In the EL, the brain's neural activity is mapped as the movement of a ball constrained by the aforementioned energy constraints. Consequently, the trajectory of this “ball” within the EL tends to roll from peaks toward basins, lingering within the basins repeatedly. Due to random fluctuations, the ball occasionally moves uphill and may transition to another basin [26].

### 3.1 Channel Selection

In EL analysis, the brain network has 2^C^ possible states at each moment, where C represents the number of channels. Because calculating the empirical distribution for each state in pMEM incurs exponential growth in fitting costs as the number of channels increases, it becomes essential to reduce the number of channels. The traditional channel selection methods can be classified into filter methods and wrapper methods. However, all these methods rely on matrix analysis, which involves flattening the original three‐dimensional fNIRS signal. They only utilize spatial or temporal information, overlooking the interaction between these two types of information. As a result, they cannot accurately characterize multidimensional fNIRS signals. Therefore, we employ tensor decomposition methods to capture the latent spatio‐temporal correlations between channels and unearth the hidden structure within the signals.

Tensor decomposition is a method used to break down a tensor into factor matrices and factor vectors, which helps avoid the loss of information caused by data flattening [53]. CPD is a classical method for dimensionality reduction of tensor data, which expresses an N‐dimensional tensor as a sum of rank‐one tensors [54]. Figure 1A illustrates the CPD.

Suppose $\chi \in {\mathbb{R}}^{F\times C\times S}$ represents the tensor of fNIRS signals for all participants, where C, S, and F, respectively, denote the number of channels, participants, and signal frequencies. The tensor can be written as

(1) $$\chi \approx \sum_{t=1}^{T}\left(a_{t}\;\circ \;b_{t}\;\circ \;d_{t}\right)$$

where *T* is a positive integer denoting the rank of the tensor. 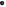 represents the inner product of vectors, and $\;\alpha^{t}\in {\mathbb{R}}^{F}$, $b^{t}\in {\mathbb{R}}^{C}$, $d^{t}\in {\mathbb{R}}^{S}$ for *t* = 1, …, *T*. Equation (1) can be converted into an elemental form as follows

(2) $$\begin{array}{c} x_{\mathrm{fcs}}\approx \sum_{t=1}^{T}\left(a_{\text{ft}}\;\circ \;b_{\mathrm{ct}}\;\circ \;d_{\mathrm{st}}\right) \end{array}$$

$$\begin{array}{c} \text{for}\;f=1,\cdots ,F,\;\;c=1,\cdots ,\;C,\;\;s=1,\cdots ,S \end{array}$$

We can integrate multiple rank‐one tensors into factor matrices, that is, $A=\left[\alpha_{1},\ldots ,\alpha_{t}\right]$, $B=\left[b_{1},\ldots ,b_{t}\right]$, and $D=\left[d_{1},\ldots ,d_{t}\right]$. Meanwhile, the factor matrices are normalized to unit length, and a weight vector $\lambda \in {\mathbb{R}}^{T}$ is introduced. Hence, the matrix form of Equation (2) is as follows.

(3) $$\begin{array}{c} \chi \approx \left[\lambda ;A,B,D\right]\equiv \sum_{t=1}^{T}\lambda_{t}\;a_{t}\;\circ \;b_{t}\;\circ \;d_{t} \end{array}$$

where $A\in {\mathbb{R}}^{F\times T}$, $B\in {\mathbb{R}}^{C\times T}$, and $D\in {\mathbb{R}}^{S\times T}$ are three factor matrices representing information from frequencies, channels, and participants, respectively.

Through the aforementioned CPD steps, we obtain the channel factor matrix B. This matrix integrates complex information regarding the interactions between the temporal, frequency, and spatial domains. Compared to traditional matrix analysis, channel selection based on CPD yields better results. We take the transpose of matrix B and conduct Pearson correlation analysis on it to obtain the correlation matrix $P\in {\mathbb{R}}^{C\times C}$. P represents the correlation between different channels. Then, P is averaged along its rows and arranged in descending order according to the mean values, resulting in the importance sequence of C channels. The CPD‐based method is applied to the four types of stimuli, and the top 7 ranked channels are extracted as the final channel sets for each stimulus.

### 3.2 Pairwise Maximum Entropy Model

[21] has demonstrated that the pMEM model based on binarized physiological signals can better simulate the dynamic transitions of brain states. Therefore, continuous fNIRS signals are binarized, which is shown in Figure 1C. For each stimulus, participant, and channel, the mean value of the fNIRS signal is used as the threshold [25]. Signals above the threshold are represented as 1; otherwise, they are represented as 0. Afterward, the activity patterns of each channel can be represented by $\left\{\sigma_{m}\left(1\right),\ldots ,\sigma_{m}\left(F\right)\right\}$, where *F* is the number of sampling points. $\sigma_{m}\left(f\right)=1$ means that the brain region corresponding to the *m*‐th channel is active at time *f*. For each participant, the brain states of the entire system are given by $s\left(f\right)=\left\{\sigma \left(1\right),\ldots ,\sigma \left(7\right)\right\}\in {\left\{-1,1\right\}}^{7}$. There are 2^7^ = 128 possible states $s_{i}$, *i* = 1, …, 128.

Firstly, pMEM calculate the empirical probability $P_{e}\left(s_{i}\right)$ of each state $s_{i}$

(4) $$\begin{array}{c} P_{e}\left(s_{i}\right)=\frac{n_{s_{i}}}{F} \end{array}$$

where $n_{\mathrm{si}}$ is the number of occurrences of state $s_{i}$ across the entire time series. ${\left\langle \sigma_{m}\right\rangle }_{e}$ denotes the empirical activation rate of *m*‐th channel, and ${\left\langle \sigma_{m}\sigma_{n}\right\rangle }_{e}$ denotes the pairwise co‐occurrence of *m*‐th and *n*‐th channels.

(5) $$\begin{array}{c} {\left\langle \sigma_{m}\right\rangle }_{e}=\frac{1}{F}\sum_{f=1}^{F}\sigma_{m}\left(f\right) \end{array}$$

(6) $$\begin{array}{c} {\left\langle \sigma_{m}\sigma_{n}\right\rangle }_{e}=\frac{1}{F}\sum_{f=1}^{F}\sigma_{m}\left(f\right)\sigma_{n}\left(f\right) \end{array}$$

Next, $P_{e}\left(s_{i}\right)$ is fitted to the Boltzmann distribution as follows.

(7) $$\begin{array}{c} P\left(s_{i}|h,J\right)=\frac{\exp\left[-E\left(s_{i}|h,J\right)\right]}{\sum_{i^{\prime }=1}^{128}\exp\left[-E\left(s_{i^{\prime }}|h,J\right)\right]} \end{array}$$

(8) $$\begin{array}{c} E\left(s_{i}\right)=-\sum_{m=1}^{7}h_{m}\sigma_{m}\left(s_{i}\right)-\frac{1}{2}\sum_{m=1}^{7}\sum_{\begin{array}{c} n=1\\ n\neq m \end{array}}^{7}J_{\mathrm{mn}}\sigma_{m}\left(s_{i}\right)\sigma_{n}\left(s_{i}\right) \end{array}$$

where $E\left(s_{i}\right)$ is the energy of $s_{i}$, and $\sigma_{m}\left(s_{i}\right)$ is the *m*‐th element of $s_{i}$. $h=\left\{h_{m}\right\}$ and $J=\left\{J_{\mathrm{mn}}\right\}$ are the parameters of the pMEM model, which are shown in Figure 1D. *h* estimates the baseline activity of all channels, while *J* estimates the interactions between pairs of channels. According to the maximum entropy theory, we obtain two equations: ${\left\langle \sigma_{m}\right\rangle }_{e}={\left\langle \sigma_{m}\right\rangle }_{\mathrm{mod}}$ and ${\left\langle \sigma_{m}\sigma_{n}\right\rangle }_{e}={\left\langle \sigma_{m}\sigma_{n}\right\rangle }_{\mathrm{mod}}$, and choose *h* and *J* that satisfy these conditions. ${\left\langle \sigma_{m}\right\rangle }_{\mathrm{mod}}$ and ${\left\langle \sigma_{m}\sigma_{n}\right\rangle }_{\mathrm{mod}}$ represent the activation rate and pairwise co‐occurrence predicted by pMEM, respectively.

(9) $$\begin{array}{c} {\left\langle \sigma_{m}\right\rangle }_{\mathrm{mod}}=\sum_{i=1}^{2^{7}}\sigma_{m}\left(s_{i}\right)P\left(s_{i}|h,J\right) \end{array}$$

(10) $$\begin{array}{c} {\left\langle \sigma_{m}\sigma_{n}\right\rangle }_{\mathrm{mod}}=\sum_{i=1}^{2^{7}}\sigma_{m}\left(s_{i}\right)\sigma_{n}\left(s_{i}\right)P\left(s_{i}|h,J\right) \end{array}$$

The pseudo‐likelihood maximization method [26] is used to estimate h and J, with a termination condition of 5 × 10^−6^ and a learning rate of 0.1.

### 3.3 Accuracy Index

The accuracy index r can measure the disparity between the results estimated by the pMEM model and the empirical data [26, 37], given by

(11) $$\begin{array}{c} r=\frac{K_{1}-K_{2}}{K_{1}} \end{array}$$

where *K*_1_ is the Kullback–Leibler divergence between empirical data and the probability distribution fitted by the independent maximum entropy model (MEM). The MEM model disregards pairwise interactions, meaning *J* = 0. *K*_2_ is the Kullback–Leibler divergence between pMEM and empirical data, which is as follows

(12) $$\begin{array}{c} K_{2}=\sum_{i=1}^{2^{7}}P_{e}\left(s_{i}\right)\log_{2}\left(\frac{P_{e}\left(s_{i}\right)}{P\left(s_{i}\right)}\right) \end{array}$$

Note that, $r\in \left[0,1\right]$ quantifies the contribution of interactions between pairs of channels. We separately employ pMEM and MEM to predict the empirical distribution and compare the differences between each of them and the empirical distribution. If the distribution fitted by pMEM matches the empirical distribution perfectly, then *r* = 1. If the disparities between the distributions generated by pMEM and MEM compared to the empirical distribution are close, then *r* = 0. In other words, pairwise interactions do not contribute to predicting the empirical distribution when *r* = 0.

### 3.4 Energy Landscape

The first step in constructing an EL is defining the adjacency relationships between states. When the Hamming distance $D_{\mathrm{mn}}$ between state $s_{m}$ and state $s_{n}$ is 1, there exists an edge $E_{\mathrm{mn}}$, which represents the transition between state m and state n. Therefore, the EL is constructed as an undirected graph composed of 2^7^ states and their corresponding energies.

We traverse through all states and their adjacent states' energy values, searching for multiple local minima (LMs) in EL [52]. The local minimum (LM) is defined as a state whose energy value is lower than the energy values of all its adjacent states. Additionally, the global minimum (GM) is also identified. For each participant, the number of LM and the energy difference between the LM and the GM are utilized as features for further analysis.

To evaluate the influence of each LM, we calculate the basin size of each LM [52]. More specifically, we randomly select an initial state $s_{m}$. If the energy value of its adjacent state $s_{n}$ is lower than that of $s_{m}$, we move to state $s_{n}$ using the gradient descent method. Otherwise, we stay in place, indicating that state $s_{m}$ is an LM. The process is repeated until reaching an LM $s_{g}$, making state $s_{m}$ belong to $s_{g}$. By traversing the 2^7^ states and executing the aforementioned procedure, each state is allocated to its corresponding LM. The basin size of an LM is defined as the number of states contained within that LM. We extract the standard deviation of the basin sizes of all LMs for each participant as the third analytical feature.

Finally, we develop a random walking model based on the EL to simulate brain neural activity. Markov chain Monte Carlo sampling is employed to simulate state transitions. We randomly select an initial state $s_{m}$, assuming that state $s_{m}$ has N neighboring states, and each of these neighboring states is chosen with equal probability. We choose a particular neighboring state $s_{n}$ with a probability of $\frac{1}{N}$. If $E\left(s_{m}\right)>E\left(s_{n}\right)$, we move to state $s_{n}$; otherwise, the probability of transition is $\exp\left[E\left(s_{m}\right)-E\left(s_{n}\right)\right]$. For each participant's EL, we conduct 20,000 iterations and calculate the duration that the system stays within the basin of the GM. This is used as the fourth EL feature in this study.

### 3.5 Method Summary

As described in section 2, fNIRS data for each participant can be categorized into four types of stimuli: calm, happy, fear, and full stimuli. For all combinations of participants and stimuli, we estimate pMEM and compare their goodness of fit to exclude the impact of model fitting differences on subsequent EL analysis. To comprehensively characterize abnormal neural activity in MDDs, we devise two approaches for EL analysis, focusing on both state‐level and individual‐level analyses. Before the statistical analysis, we perform the Shapiro–Wilk test [55] on all data. The results show that, except for the number of LM, the remaining data conform to a normal distribution. Therefore, we choose the Kruskal–Wallis nonparametric test [56] to analyze the number of LM, while the remaining data are analyzed using analysis of variance (ANOVA) and Tukey's post hoc tests.

1. EL analysis based on major states: although the LM sets differ for each combination, the 120 participants all choose the same two LMs (0000111 and 1111000) across four stimulus categories, which we refer to as major states. The durations within the basins of major states are exacted for ANOVA and Tukey's post hoc tests between groups and stimuli.
2. EL analysis based on participants: four energy features are used for statistical analysis (Section 3.4). We use the Kruskal–Wallis test for the number of LM and ANOVA with Tukey's post hoc tests for energy differences, basin sizes, and the duration of GM.

Finally, we train two classification models using the connectivity features and energy features separately. For each participant, the connectivity feature vector is composed of the *J* values from the pMEM, while the energy feature is a concatenated vector of the energy of 2^7^ states, the number of LM, energy differences, basin sizes, and the duration of GM. The MinMaxScaler method is used for data standardization. A support vector machine (SVM) is employed for classification, with the following parameters: kernel = “rbf,” tol = 0.001, class weight = “balanced,” gamma = 10^−8^~10^8^, C = 10^−8^~10^8^. We use grid search to select the optimal gamma and C, and leave‐one‐out cross‐validation to evaluate the predictive abilities of the models.

## 4 Experiment and Results

### 4.1 Channel Selection

**FIGURE 2:**
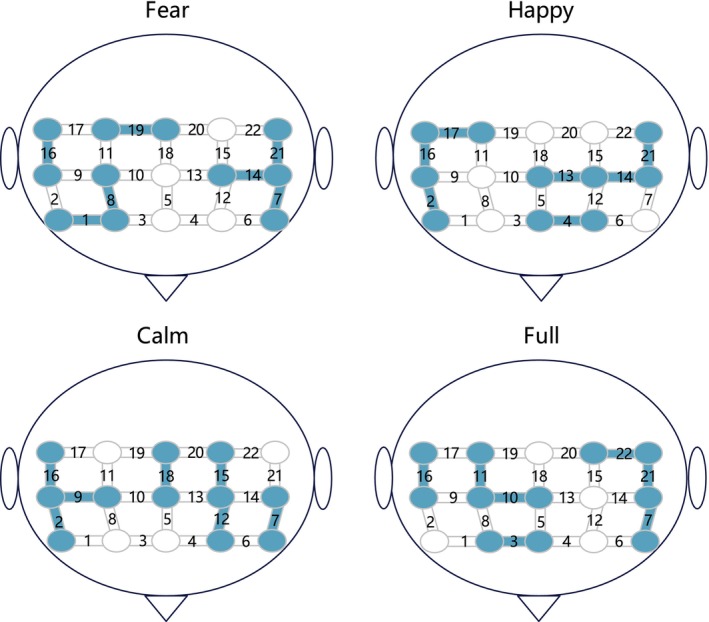
Channels selected through tensor decomposition method.

Figure 2 displays the channels selected under four types of stimuli, which exhibit a high degree of similarity. The selected channels are predominantly concentrated in the medial frontal gyrus (MFG) region, including channels 2, 9, 7, 14, 16, 21, and 22. This finding is consistent with previous research, indicating that, irrespective of the emotional stimuli, the activation levels in the MFG are consistently higher in MDDs compared to HCs [57, 58]. We believe that channels located within the MFG might possess more discriminative features and spatio‐temporal correlations, hence being repeatedly selected.

### 4.2 Accuracy Index of pMEM

We construct a pMEM model for each participant and evaluate the model's goodness of fit using the coefficient r. The fitting results reveal a high accuracy in estimating the empirical distribution by pMEM, with a mean of 0.851 and a standard deviation of 0.083 for the coefficient r. ANOVA is used to assess the differences in r among pMEMs. There are no significant differences between groups (*F*(1, 472) = 2.849, *p* = 0.092), but significant main effects exist among stimuli (*F*(3, 472) = 160.547, *p* < 0.001). This indicates that the changes in neural activity across different types of stimuli influence the goodness of fit of pMEMs in participants. The results of Tukey's post hoc test show a significant difference between full and calm (*p* < 0.001), happy and calm (*p* = 0.048), full and fear (*p* < 0.001), and full and happy (*p* < 0.001).

### 4.3 Energy Landscape Analysis Based on Major States

**FIGURE 3:**
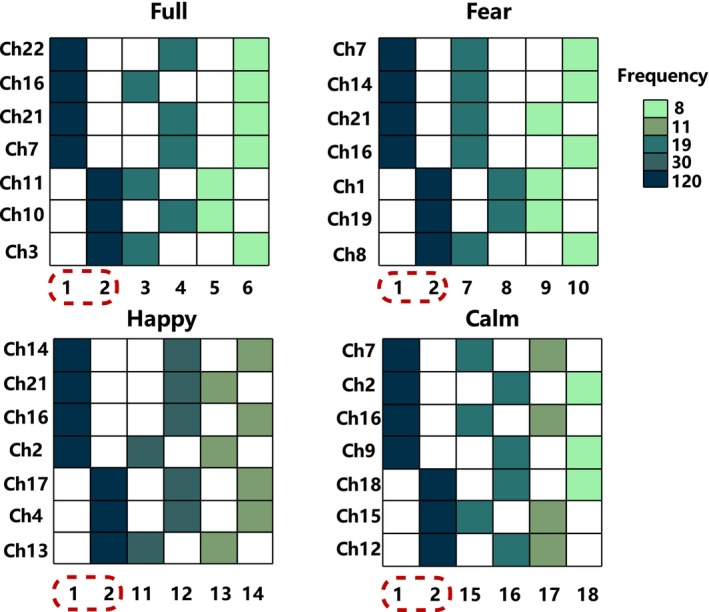
High‐frequency LM states under different stimuli. Horizontal numbers represent the state numbers, and vertical numbers represent the seven channel numbers retained after channel selection for each stimulus. Filled cells indicate activation of the corresponding channels, while blank cells represent the opposite. Colors are used to distinguish the frequency of occurrence of each state. The red dashed lines mark two major states.

We categorize all EL models into four stimuli, with each stimulus comprising 120 participants' ELs. Each EL contains several LMs. In general, the sets of LMs for each EL are distinct. However, some states contain rich information, leading them to be selected as LM by multiple ELs. We compile the six most frequently occurring LMs for each stimulus, assigning them numbers 1~18, as shown in Figure 3. Filled and blank cells, respectively, represent whether the corresponding channel is activated (denoted as 1) or not activated (denoted as 0) in that LM. The filled color indicates the frequency of the state being chosen as an LM. Taking major state 1 under full stimuli as an example, it is denoted as 1111000, indicating that ch22, ch16, ch21, and ch7 are activated, while ch11, ch10, and ch3 are abandoned. The dark color indicates that it is chosen as an LM by all 120 ELs.

The experimental results reveal that even under different stimuli, States 1 and 2 (1111000 and 0000111) are present in the LM sets of all participants, and we refer to them as major states. We also observe that although the selected channels differ under each stimulus, channels activated by major state 1 under all four stimuli belong to the MFG region, while channels activated by major state 2 belong to the SFG region. We extract the average duration of the major states to explore differences between groups and stimuli.

**FIGURE 4:**
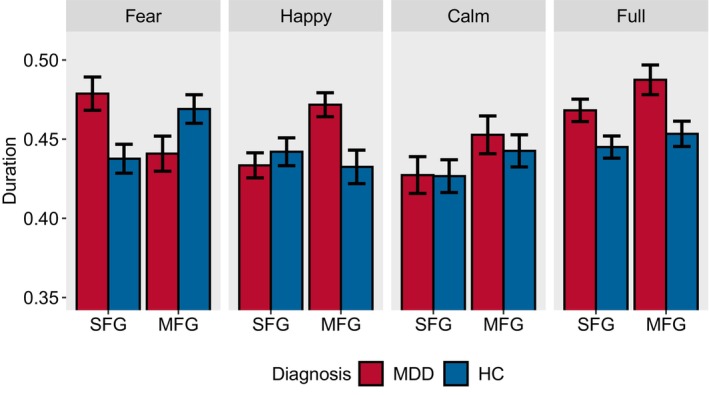
The average duration of major states under each stimulus. MFG (Middle Frontal Gyrus) and SFG (Superior Frontal Gyrus), respectively, represent the regions where major States 1 and 2 are located (HCs, healthy controls; MDDs, individuals with MDD).

We first present the distribution of the average duration using a dot plot (Figure S3), followed by a bar chart (Figure 4) to display the mean and standard deviation of the data. In Figure 4, it is noted that the duration of the two major states in MDDs under full stimuli is greater than that in HCs. When facing negative stimuli, MDDs show longer duration in the SFG region than HC and shorter duration in the MFG region. Under positive stimuli, MDDs exhibit shorter duration than HCs in the SFG region, while in the MFG region, it is the opposite. The inter‐group changes induced by neutral stimuli are similar to those induced by full stimuli. We believe these differences are related to abnormal neural activity in the brains of MDDs when facing different stimuli. The specific details will be explained in section 5.1.

We conduct separate ANOVA tests for the duration of the two major states. There are significant differences in the duration of major state 1 between groups (*F*(1, 472) = 3.975, *p* = 0.047) and group: stimuli (*F*(3, 472) = 4.914, *p* = 0.002). The difference between the stimuli is not statistically significant (*F*(3, 472) = 2.032, *p* = 0.109). Similarly, the duration of major state 2 exhibits significant main effects for stimuli (*F*(3, 472) = 5.419, *p* = 0.001), groups (*F*(1, 472) = 4.708, *p* = 0.031) and group: stimuli (*F*(3, 472) = 2.975, *p* = 0.031). The Tukey's post hoc test is employed for further analysis. In the case of major state 1, there are significant differences between full and calm (*p* = 0.049). In the case of major state 2, the interactions of the following stimuli are significant: fear‐calm (*p* = 0.004) and full‐calm (*p* = 0.007).

### 4.4 Energy Landscape Analysis Based on Participants

Figure S4 shows the distribution of four energy features under different stimuli, with the number of LM does not exhibit a normal distribution trend, while others approximately following a normal distribution.

**FIGURE 5:**
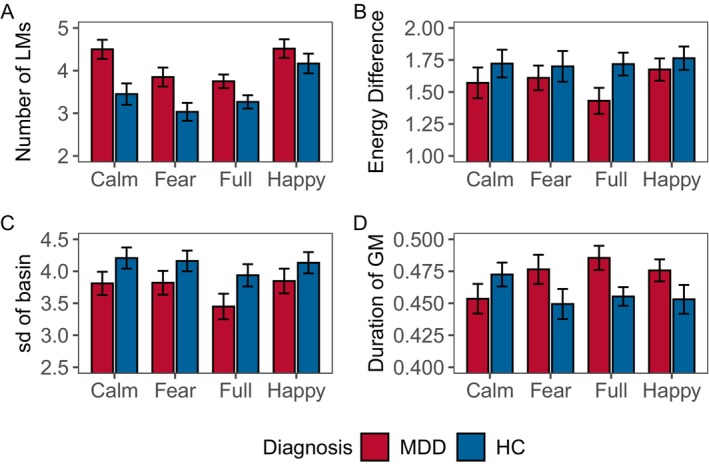
Energy features of all participants. (A) number of LMs, (B) mean energy difference between LMs and GM, (C) standard deviation of basin sizes, and (D) duration of GM. The bars graph show the mean value, and the error bars shows the 95% confidence interval for the mean value (HCs, healthy controls; MDDs, Individuals with MDD).

All types of stimuli show that the number of LMs is greater in MDDs than in the HCs ($\chi^{2}$ (1) = 22.368, *p* < 0.001) (Figure 5A). Additionally, there are significant differences between stimuli ($\chi^{2}$ (3) = 18.780, *p* < 0.001).

For each combination of stimuli and participants, the average energy difference between LMs and GM is used as the second indicator (Figure 5B). HCs consistently exhibit a larger energy difference than MDDs (*F*(1, 472) = 4.521, *p* = 0.034). No significant main effects between stimuli (*F*(3, 472) = 0.674, *p* = 0.568) and group: stimuli (*F*(3, 472) = 0.416, *p* = 0.742).

The HCs uniformly have a larger standard deviation in basin size (*F*(1, 472) = 8.902, *p* = 0.003) (Figure 5C). No significant differences between stimuli (*F*(3, 472) = 1.441, *p* = 0.230). The interaction between group and stimuli is not statistically significant (*F*(3, 472) = 0.117, *p* = 0.950).

The duration of the GM shows significant differences in the group (*F*(1, 472) = 4.267, *p* = 0.039) and group: stimuli (*F*(3, 472) = 2.648, *p* = 0.048) (Figure 5D). No significant main effects between stimuli (*F*(3, 472) = 0.343, *p* = 0.794).

### 4.5 Comparison Between pMEM Features and Energy Features

For the depression detection model based on connectivity features and energy features, we compare the classification abilities of the two. We extract the accuracy, recall, precision, and *F*1 score to visualize the differences between the two models, as shown in Table 2.

**TABLE 2:** The comparative results using the SVM classifier.

			Acc	Pre	Rec	Spec	F1
Connectivity	Fear	Mean	72.46	74.41	74.31	74.46	0.72
		Std.	12.66	10.19	11.89	13.54	0.12
	Happy	Mean	65.12	67.82	68.43	67.38	0.63
		Std.	11.63	12.48	11.09	12.31	0.12
	Calm	Mean	58.92	62.48	61.62	62.86	0.59
		Std.	9.77	14.80	15.44	14.97	0.10
	Full	Mean	64.05	68.54	65.68	68.34	0.64
		Std.	10.62	9.76	12.01	11.73	0.12
Energy	Fear	Mean	**86.53**	**86.76**	**88.30**	**86.73**	**0.87**
		Std.	8.71	7.85	9.47	10.54	0.08
	Happy	Mean	76.11	76.94	79.44	77.18	0.76
		Std.	10.01	12.23	10.63	9.81	0.10
	Calm	Mean	72.67	73.43	75.98	73.52	0.72
		Std.	13.91	9.08	11.38	12.22	0.13
	Full	Mean	81.30	81.44	82.64	82.45	0.81
		Std.	8.17	10.58	11.69	12.38	0.11

*Note:* Bold values indicate the best classification performance in each column.

Abbreviations: Acc, accuracy; F1, F1 score; Pre, precision; Rec, recall; Spec, specificity.

The results indicate that the model based on energy features exhibits superior classification performance. All parameters show significant differences between models (accuracy: *F*(1, 792) = 228.389, *p* < 0.001, precision: *F*(1, 792) = 75.310, *p* < 0.001, recall: *F*(1, 792) = 126.927, *p* < 0.001, specificity: *F*(1, 792) = 85.044, *p* < 0.001, *F*1 score: *F*(1, 792) = 199.249, *p* < 0.001), and stimuli (accuracy: *F*(3, 792) = 35.820, *p* < 0.001, precision: *F*(3, 792) = 19.556, *p* < 0.001, recall: *F*(3, 792) = 16.093, *p* < 0.001, specificity: *F*(1, 792) = 16.333, *F*1 score: *F*(3, 792) = 36.084, *p* < 0.001). This suggests that on the basis of simulating brain activity with pMEM, conducting EL analysis is essential. By extracting features from EL to characterize LMs, transforming neurodynamics into attractor dynamics can significantly enhance the capabilities of the depression detection model.

Tukey's post hoc tests reveal significant differences among fear and calm (accuracy: *p* < 0.001, precision: *p* < 0.001, recall: *p* < 0.001, specificity: *p* < 0.001, *F*1 score: *p* < 0.001), full and calm (accuracy: *p* < 0.001, precision: *p* < 0.001, recall: *p* = 0.014, specificity: *p* < 0.001, *F*1 score: *p* < 0.001), full and fear (accuracy: *p* < 0.001, precision: *p* = 0.002, recall: *p* < 0.001, specificity: *p* = 0.028, *F*1 score: *p* < 0.001), happy and calm (accuracy: *p* = 0.016, precision: *p* = 0.049, recall: *p* = 0.021, specificity: *p* = 0.011, *F*1 score: *p* = 0.002), as well as happy and fear (accuracy: *p* < 0.001, precision: *p* < 0.001, recall: *p* < 0.001, specificity: *p* < 0.001, *F*1 score: *p* < 0.001) in four classification metrics. Among them, the fear stimulus segment exhibits the highest classification performance, with an accuracy of 86.53% and a recall of 88.30%. This may be related to the negative bias observed in MDDs.

## 5 Discussion

In this study, we divide the fNIRS data of each participant into four stimulus segments and construct EL for each stimulus of each participant to characterize the neural dynamics of MDDs. For all 480 ELs, we conduct analyses based on both major states and participants. The statistical results reveal that the number of LMs, basin size, duration of GM, and the energy difference between LMs and GM can serve as significant indicators for MDDs. We also attempt to relate energy features to the mechanisms of depression and find that these analysis results are highly correlated with existing studies on depressive symptoms, such as negative emotional bias, rumination, and restricted brain networks.

### 5.1 State‐Level Analysis and Depressive Symptoms

Given that this study employs an affective stimulus paradigm, we initially explore whether there is a connection between ELs and emotional abnormalities. Current research suggests an association between the negative emotions in MDDs and abnormalities in local cerebral blood flow [59]. Additionally, both positive and negative thinking are associated with the activation of specific brain regions [60]. These results imply that the emotional symptoms in MDDs may serve as a plausible explanation for the findings of EL analysis.

Negative emotional bias is one of the endophenotypes of depression, referring to the tendency of MDDs to exhibit preferential attention to negative information and impaired inhibition [61]. Negative emotional bias leads to intensified processing of negative information in MDDs, and MDDs tend to interpret positive stimuli as negative experiences, thereby perpetuating depression [62]. Past research has already demonstrated a strong association between negative emotional bias and depression. When exposed to positive stimuli, MDDs show increased activation in the SFG region and decreased activation in the MFG region [63]. Conversely, when facing negative stimuli, the pattern is reversed, with decreased activation in the SFG region and increased activation in the MFG region [64]. We will further provide robust evidence for this association through EL features.

In Section 4.3, we extract major states 1 and 2, representing the activation of channels in the MFG and SFG, respectively. The duration in Figure 4 represents the time that the brain spends in the basins of the major states during activity, quantifying the influence of the major states on the brain's dynamic network. Using the fear stimuli as an example, let's illustrate the relationship between duration and regional activation. When MDDs listen to negative audio, the duration in the SFG region is longer than in the HCs. This indicates that the small ball randomly wandering in the EL is more likely to be captured by the SFG region and is difficult to break free from its control. The ball staying in the same state basin for an extended period prevents it from switching states frequently. This indicates a less active network in the SFG region, corresponding to a decreased activity frequency and reduced activation in the SFG area. Therefore, the level of regional activation is inversely proportional to the duration of the corresponding state in that region.

From this, we can further interpret the experimental results of Figure 4. Compared to HCs, MDDs show a longer duration of activation in the SFG region and a shorter duration in the MFG region during negative stimuli, which suggests reduced activation in the SFG region and increased activation in the MFG region. In the case of positive stimuli, the opposite results in duration imply that the activation levels are also opposite. They are consistent with previous research findings related to negative emotional bias.

In full stimuli and calm stimuli, MDDs exhibit longer durations in both brain regions, suggesting the brain is trapped in a single basin, unable to escape. This appears similar to the rumination behavior of MDDs, where they immerse themselves in the emotions of pain and worry, engaging in repetitive self‐analysis [65]. In response to external stimuli, rumination is marked by reduced activity in the prefrontal cortex region [66], particularly the dlPFC (equivalent to MFG) [67]. Based on the previous conclusions, inactive networks imply longer durations; thus, the rumination characteristics align with the inter‐group comparisons across full stimuli and calm stimuli.

### 5.2 Individual‐Level Analysis and Depressive Symptoms

In this section, we attempt to interpret the results of the EL analysis based on participants in Section 4.4. Figure 5A indicates that MDDs generally have more LMs than HCs. LMs, as attractors that frequently appear in state transitions, can be considered as constraints within the network. The network constraints uncovered in this experiment might reveal a general lack of flexibility in the response systems of MDDs [68], indicating that the brain's degrees of freedom are fewer in MDDs. This discovery may be associated with the pathology of MDDs, specifically, structural alterations in the basal ganglia circuits, characterized by smaller volumes in the bilateral caudate nucleus and putamen [69, 70]. We hypothesize that the neurodegenerative process linked to the atrophy of the caudate nucleus might lead to the doping of many interfering LMs in addition to normally functioning LMs [71]. These additional LMs might result in information interruptions, impeding the normal transmission of information.

GM is the most influential attractor among all LMs, shaping the overall structure of the EL. The energy difference between GM and LMs suggests the degree of ease in transition between the two. The larger the energy difference, the more likely a state transition is to occur. We compute the average of all differences (Figure 5B). MDDs exhibit smaller energy differences, that is, fewer state transitions, implying lower degrees of freedom in their brains.

Similarly, GM possesses the largest basin in the EL, thereby exerting significant influence on the variation in the basin sizes of LMs. We calculate the standard deviation of the basin sizes for each LM to measure the variation in basin sizes, essentially quantifying the similarity between the basins of LMs and GM (Figure 5C). The basins in MDDs display smaller variations, signifying a greater similarity between LM and GM. This finding needs to be jointly explained with the number of LMs. In the definition of basin size, each state can only be assigned to one basin, and the size of each basin is equal to the number of states it contains. Due to the constant total number of states, when there are more LMs in the EL, their basins will be smaller. In conclusion, MDDs have mutually similar small basins, which possess stronger attractor characteristics [25]. This reveals that the ELs of MDDs have stronger constraints, consistent with the earlier conclusions.

Finally, we analyze the duration of the simulated random walk system in the GM. Except for positive stimuli, MDDs spend a longer time in the GM basin in the remaining three conditions (Figure 5D). This further confirms that MDDs are constrained by the strong GM basin, resulting in a prolonged stay of the system within the GM basin. On the contrary, HCs experience a large yet weak attraction from GM, allowing them to more easily escape the influence of GM. This result once again provides compelling evidence for the restricted and disrupted brain information transmission in MDDs.

### 5.3 Limitations and Challenges

This study discusses the neurodynamic changes in MDDs, but there are still limitations and challenges that need to be addressed. First, EL analysis requires binarization of the continuous fNIRS signals, which leads to a certain degree of information loss. Second, this study does not achieve a highly accurate pMEM model, with an average *r*‐value of 0.851. This may be due to the low sampling rate of the fNIRS device, resulting in a limited number of usable data points per recording, which is insufficient to meet the requirements for fitting a more precise pMEM model. Finally, the computational cost of pMEM increases exponentially with the number of channels, so we are only able to select 7–10 channels for model fitting. Data from most channels are not included in the EL analysis, leading to data wastage, which may have further impacted the accuracy of the pMEM model. In future research, the effects of different thresholds on the experimental results can be discussed to select the optimal threshold and minimize the impact of binarization on the signals. Additionally, increasing the duration of the experimental paradigm could enhance the amount of information in each fNIRS recording, allowing for a higher‐precision pMEM model.

## 6 Conclusion

In this study, we customize a data‐driven EL model for fNIRS signals, enabling the observation of the brain dynamics in MDDs while eliminating artificial interference. There are significant differences in the duration of major states, the number of LMs, the energy difference between GM and LMs, the standard deviation of basin size, and the duration of GM between MDDs and HCs, which indicate that MDDs imply constrained and rigid characteristics in their brain dynamic networks. We further explore potential connections between experimental results and symptoms of depression such as rumination and negative emotional bias. In addition, we demonstrate that pMEM and EL features can be utilized for depression detection. These results suggest that EL might have potential applications in the clinical diagnosis of depression, providing a novel perspective for the study of its pathological mechanisms.

## Author Contributions

Yushan Wu led the experiment design and drafted the manuscript. Shi Qiao, Jitao Zhong, and Lu Zhang assisted with experimental procedures and data collection. Bin Hu contributed to the research design and methodology. Juan Wang focused on data interpretation, particularly in psychiatric aspects. Hong Peng, the corresponding author, coordinated the research, interpreted the results, and finalized the manuscript.

## Conflicts of Interest

The authors declare no conflicts of interest.

We report how we determined our sample size, all data exclusions, all manipulations, and all measures in the study. Data are analyzed using R, version 4.3.1.

## Supporting information


Figure S1.
Figure S2.
Figure S3.
Figure S4.

## Data Availability

The datasets used and analyzed during the current study are available from the corresponding author upon reasonable request.
