# Supplementary material for: FNIRS‐Based Energy Landscape Analysis to Signify Brain Activity Dynamics of Individuals With Depression
Source: CNS Neurosci Ther. 2024 Dec 1;30(11):e70139. doi: 10.1111/cns.70139 (PMC11609116; doi:10.1111/cns.70139)
Supplement: Supplementary file 1 — Figure S1. Figure S2. Figure S3. Figure S4. [file CNS-30-e70139-s001.zip › supplementary materials.docx]

**Supplementary materials**


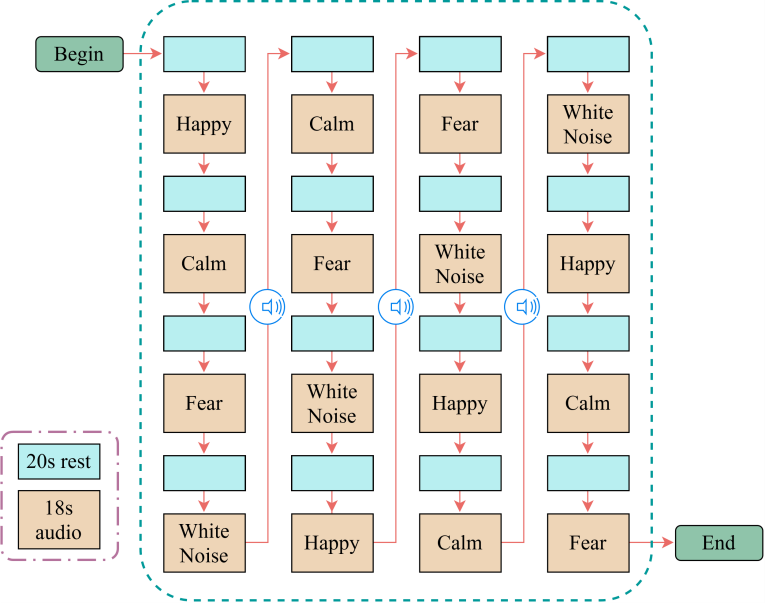


**Figure S1.** Diagram of the experimental paradigm based on auditory stimuli. Specifically, participants wear fNIRS measurement headbands and complete four sets of audio tests. Each test set comprises four types of stimuli: positive, neutral, negative, and white noise.


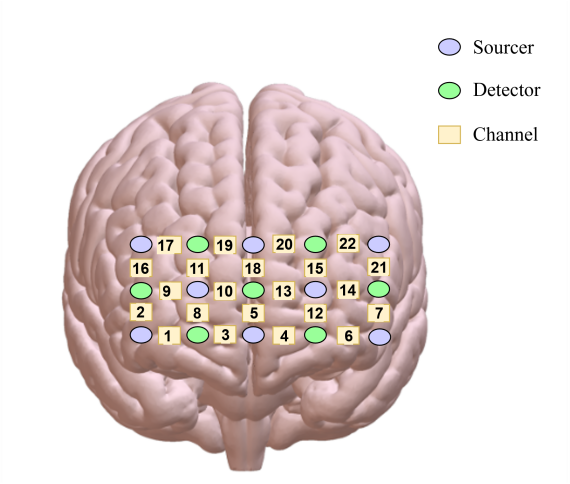


**Figure S2.** The arrangement of fNIRS optodes on the prefrontal cortex region. The purple and green ellipses respectively represent sources and detectors. There are a total of 22 channels generated between 7 emitters and 8 detectors.


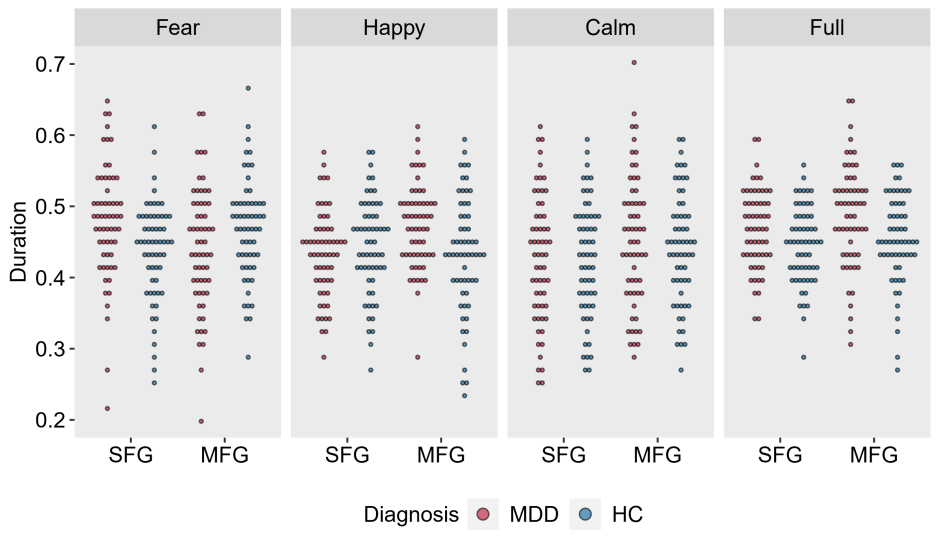


**Figure S3.** The dot plot of the average duration for all participants under different stimuli, where MFG (Middle Frontal Gyrus) and SFG (Superior Frontal Gyrus) are associated with major state 1 and 2, respectively (MDDs: individuals with MDD, HCs: healthy controls).


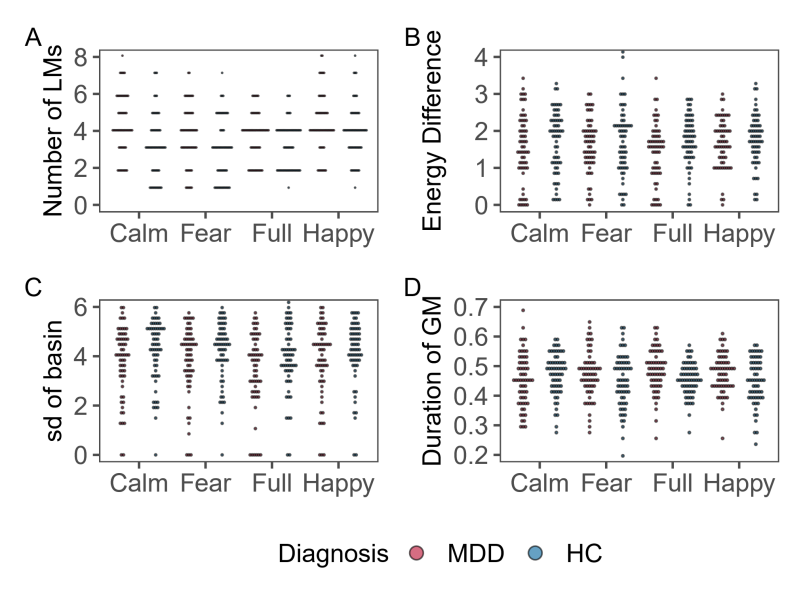


**Figure S4.** The dot plot of four energy features for all participants. (A) number of LMs, (B) mean energy difference between LMs and GM, (C) standard deviation of basin sizes, and (D) duration of GM (MDDs: individuals with MDD, HCs: healthy controls).
