# Supplementary figures and images for: FNIRS‐Based Energy Landscape Analysis to Signify Brain Activity Dynamics of Individuals With Depression
Source: CNS Neurosci Ther. 2024 Dec 1;30(11):e70139. doi: 10.1111/cns.70139 (PMC11609116; doi:10.1111/cns.70139)

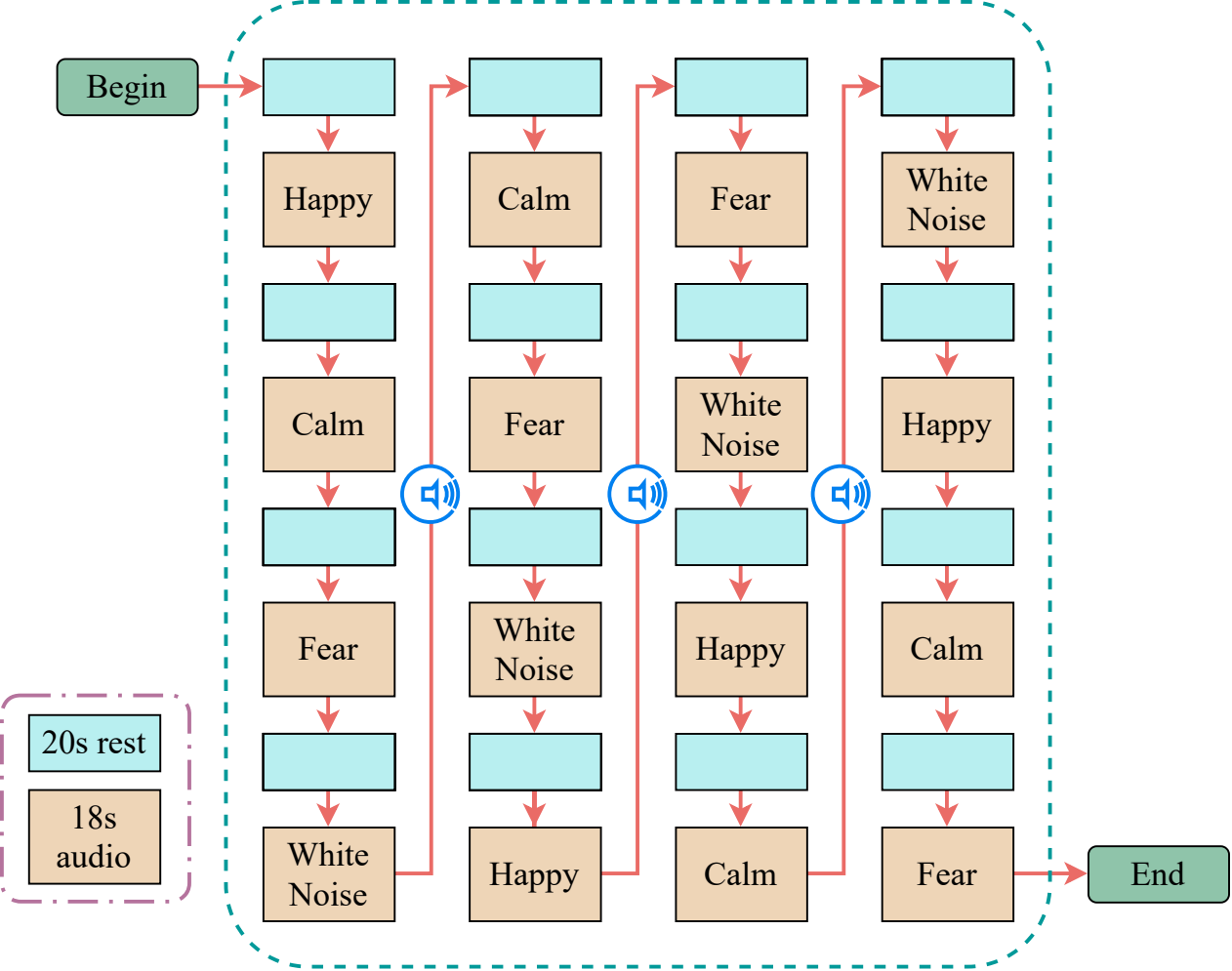

○ Sourcer

● Detector

□ Channel

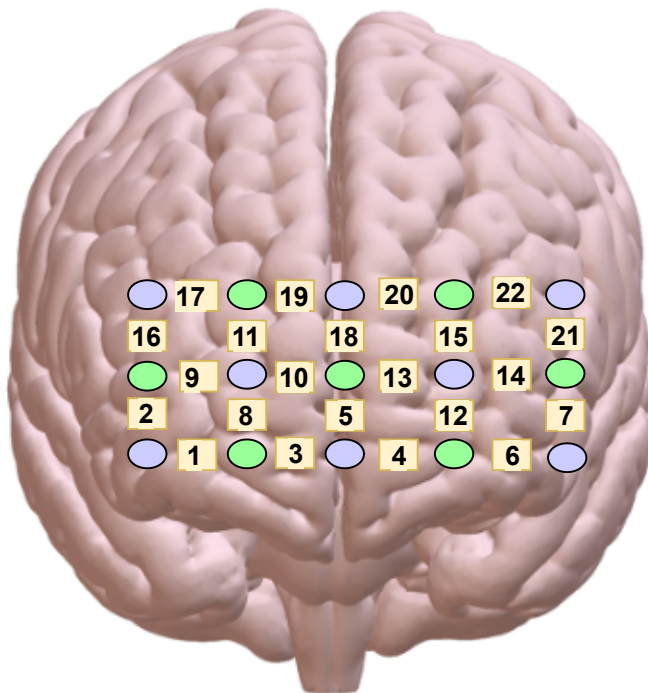

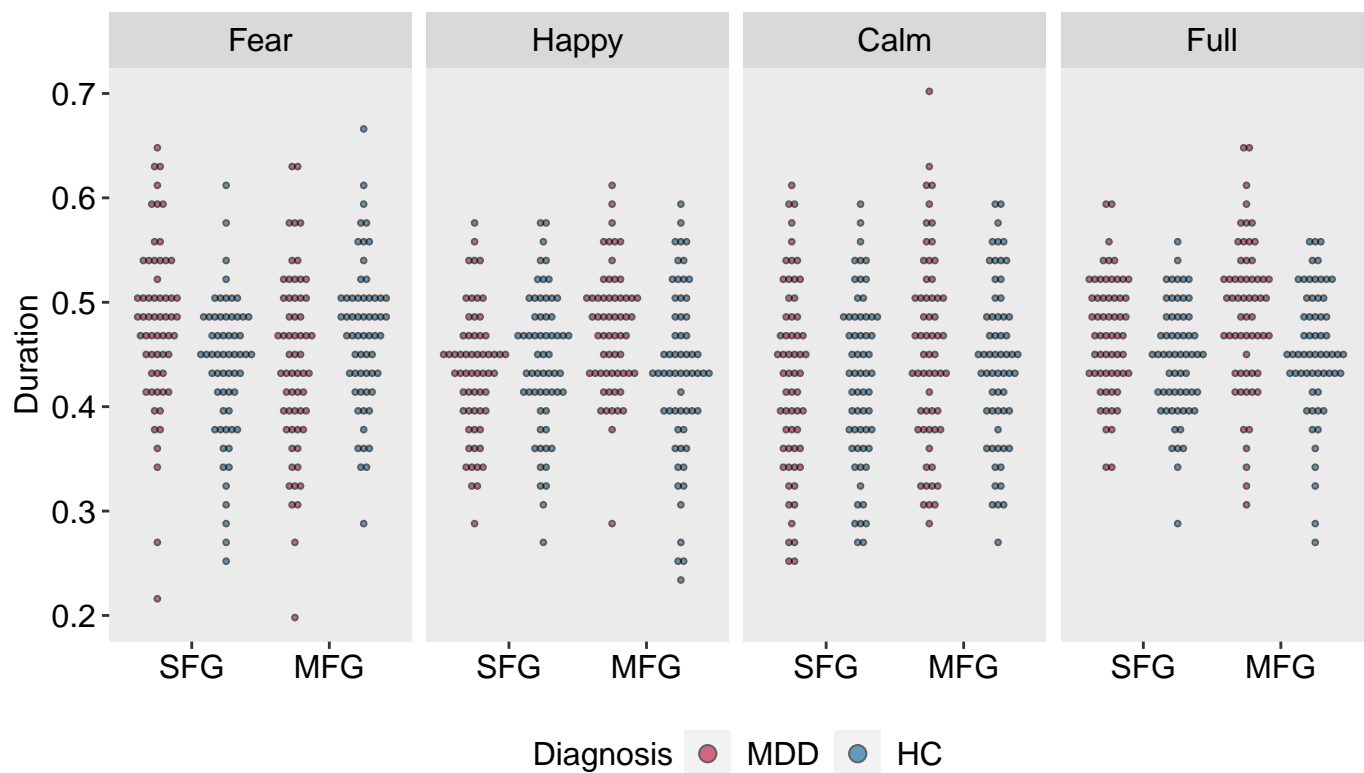

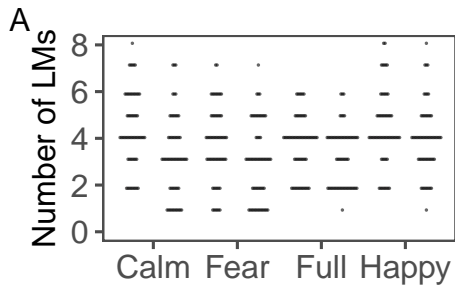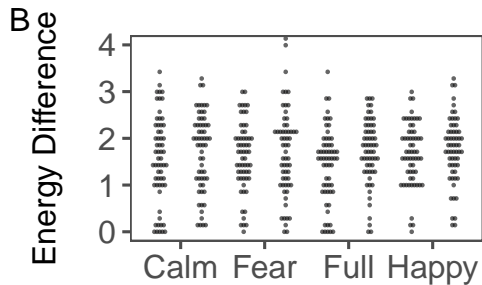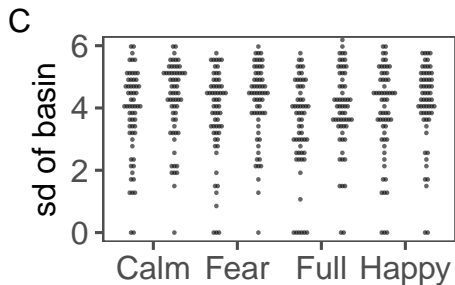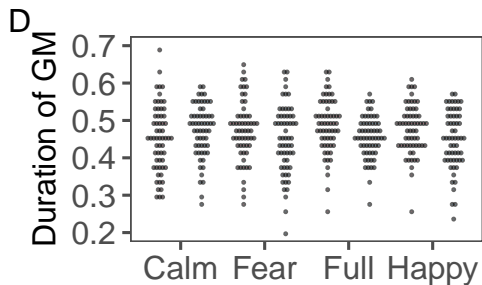

Supplement: Supplementary file 1 — Figure S1. Figure S2. Figure S3. Figure S4. [file CNS-30-e70139-s001.zip › All unedited plot of supplementary materials.pdf]
